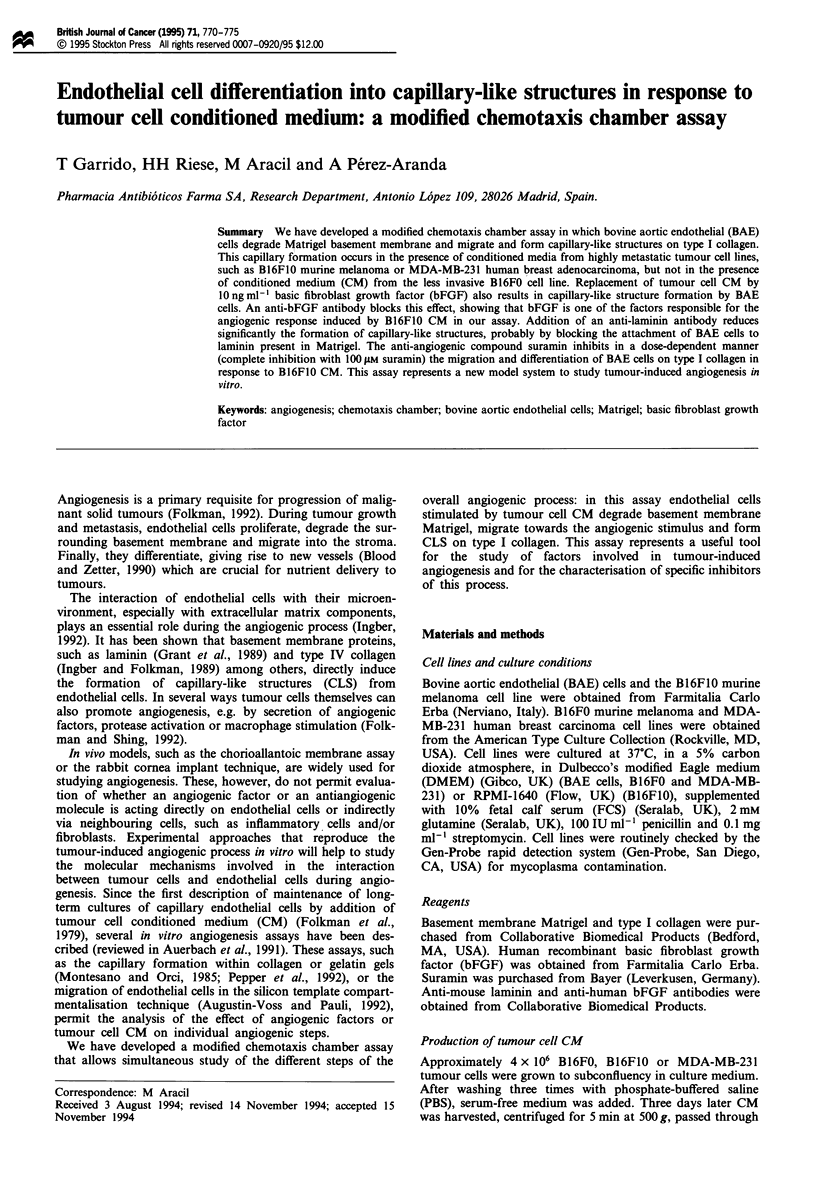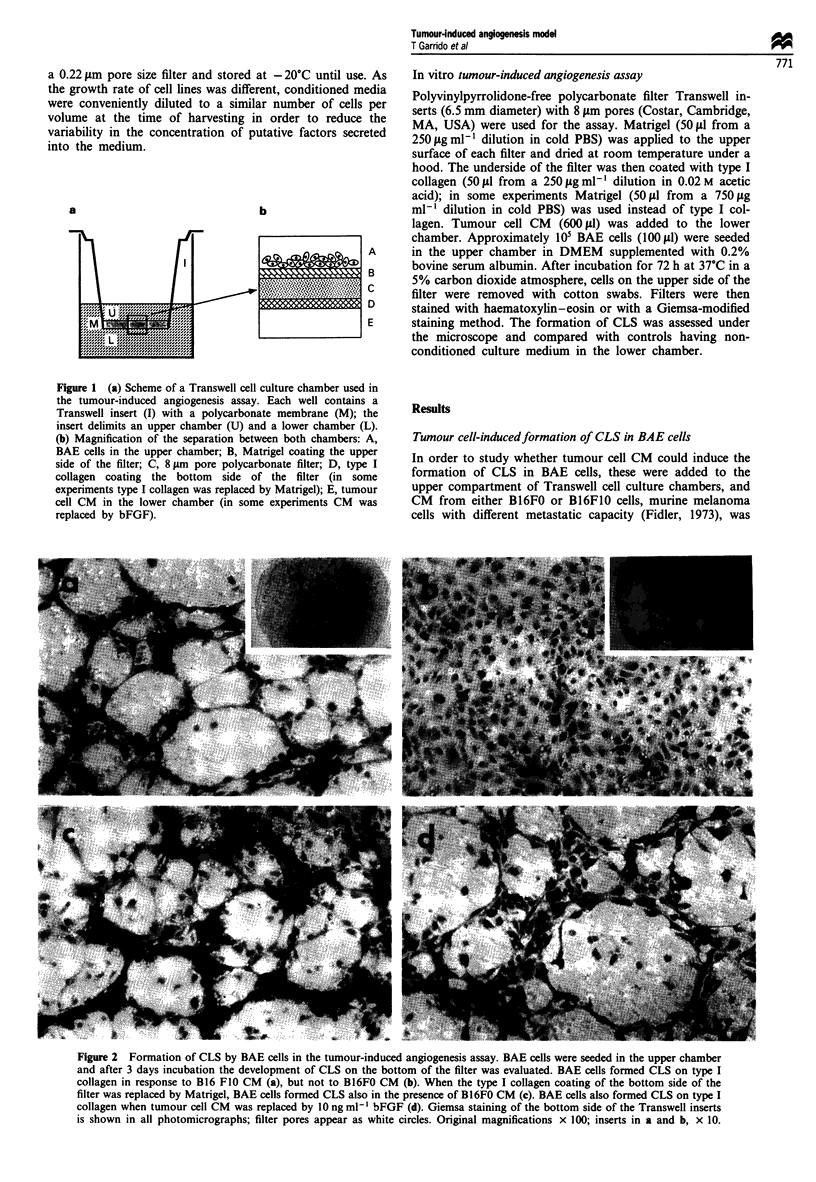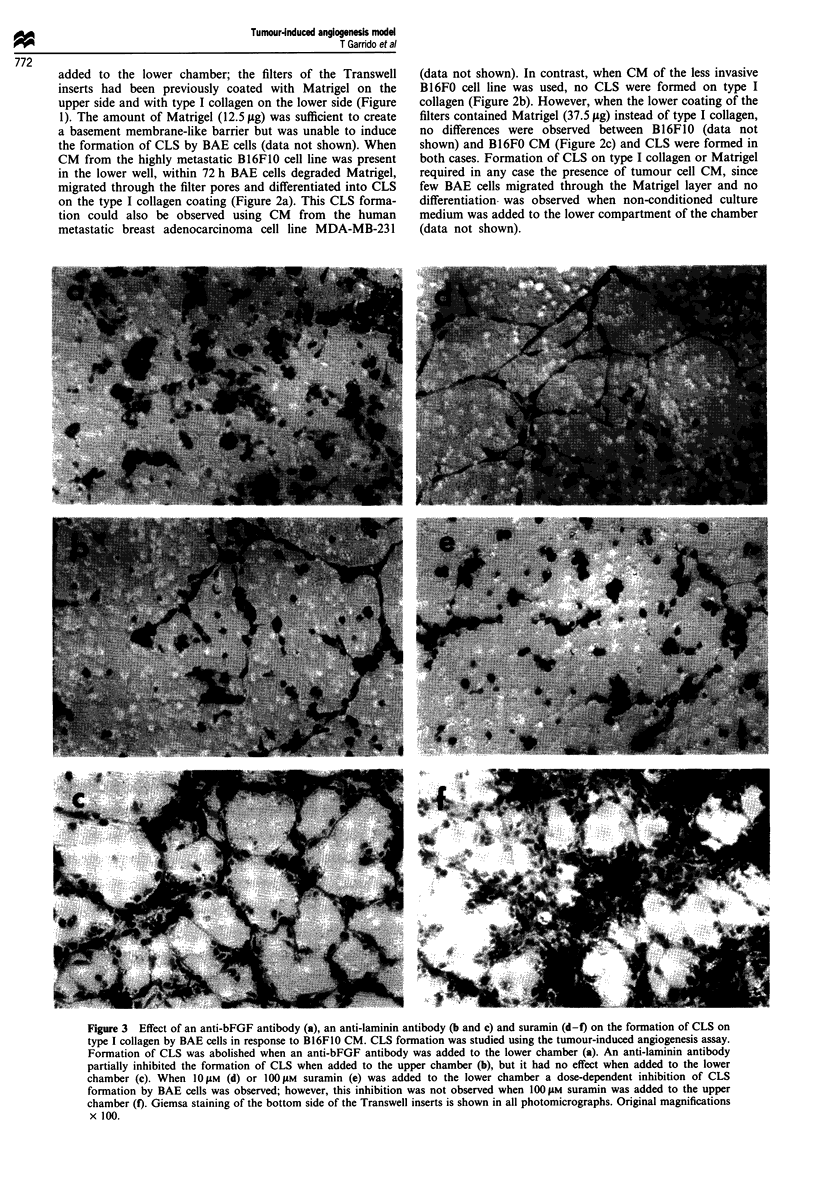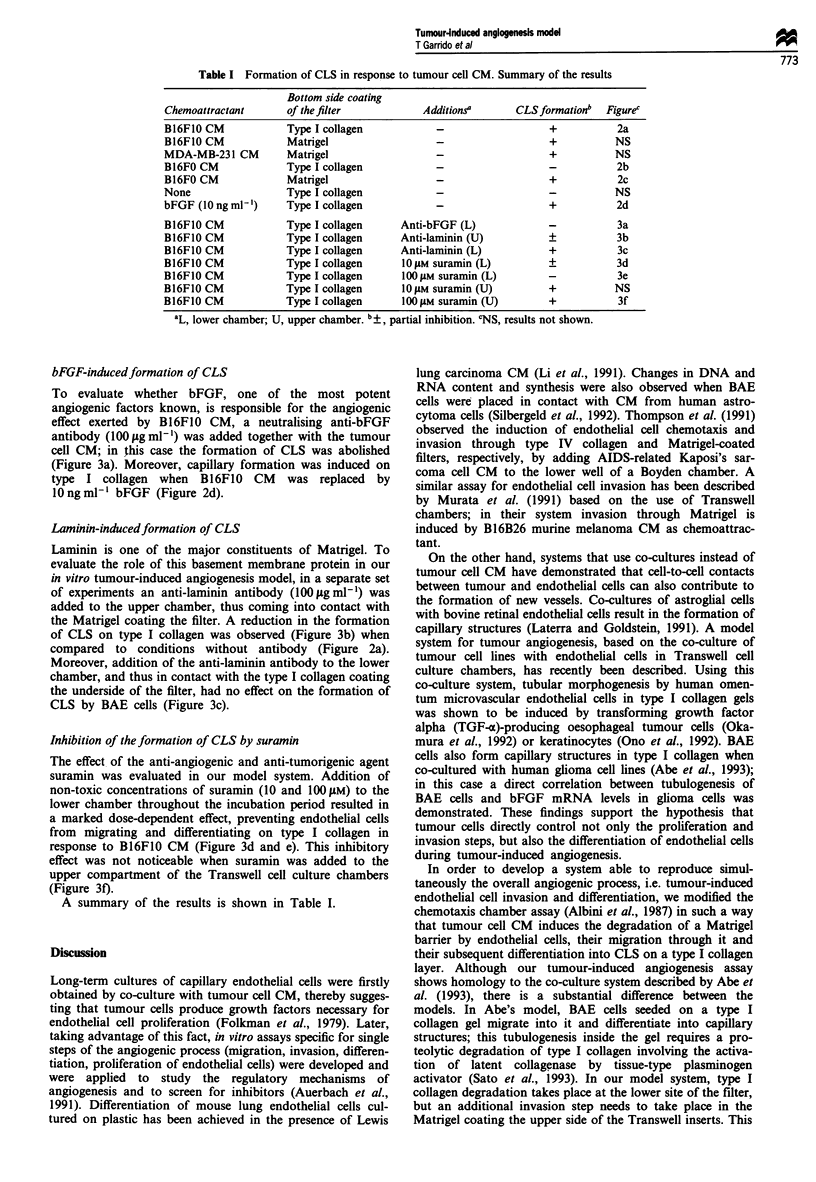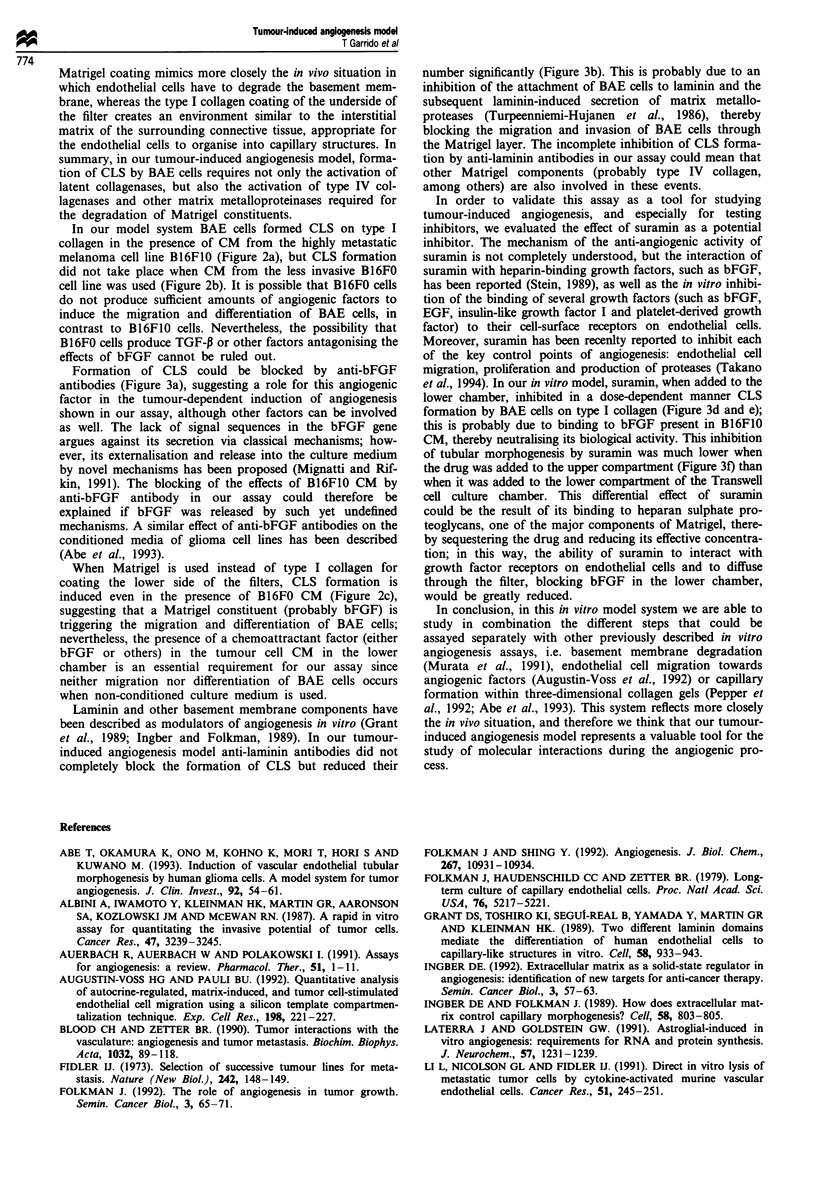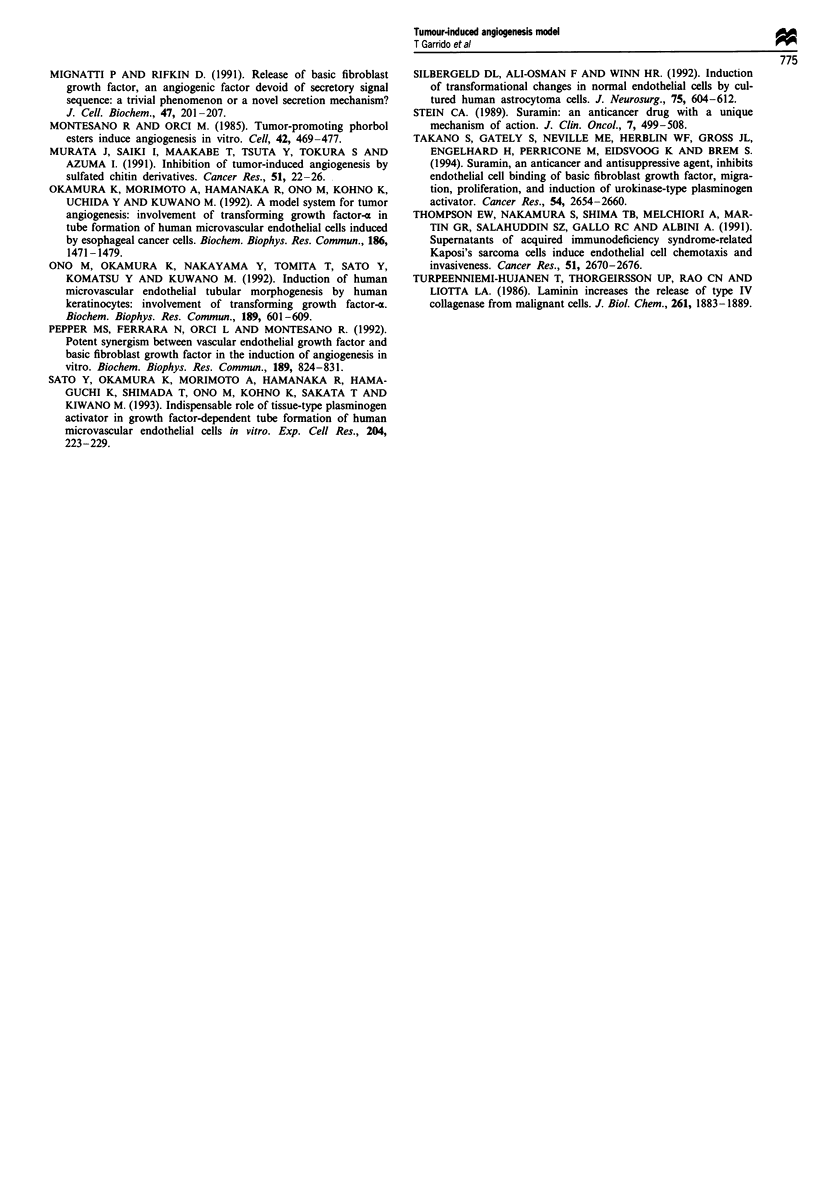# Endothelial cell differentiation into capillary-like structures in response to tumour cell conditioned medium: a modified chemotaxis chamber assay.

**DOI:** 10.1038/bjc.1995.149

**Published:** 1995-04

**Authors:** T. Garrido, H. H. Riese, M. Aracil, A. Pérez-Aranda

**Affiliations:** Pharmacia Antibióticos Farma SA, Research Department, Madrid, Spain.

## Abstract

**Images:**


British Journal of Cancer (1995) 71, 770-775
© 1995 Stockton Press All rights reserved 0007-0920/95 $12.00

### Endothelial cell differentiation into capillary-like structures in response to tumour cell conditioned medium: a modified chemotaxis chamber assay

T Garrido, HH Riese, M Aracil and A Pérez-Aranda

*Pharmacia Antibióticos Farma SA, Research Department, Antonio López 109, 28026 Madrid, Spain.*

**Summary** We have developed a modified chemotaxis chamber assay in which bovine aortic endothelial (BAE) cells degrade Matrigel basement membrane and migrate and form capillary-like structures on type I collagen. This capillary formation occurs in the presence of conditioned media from highly metastatic tumour cell lines, such as B16F10 murine melanoma or MDA-MB-231 human breast adenocarcinoma, but not in the presence of conditioned medium (CM) from the less invasive B16F0 cell line. Replacement of tumour cell CM by 10 ng $ml^{-1}$ basic fibroblast growth factor (bFGF) also results in capillary-like structure formation by BAE cells. An anti-bFGF antibody blocks this effect, showing that bFGF is one of the factors responsible for the angiogenic response induced by B16F10 CM in our assay. Addition of an anti-laminin antibody reduces significantly the formation of capillary-like structures, probably by blocking the attachment of BAE cells to laminin present in Matrigel. The anti-angiogenic compound suramin inhibits in a dose-dependent manner (complete inhibition with 100 $\mu$M suramin) the migration and differentiation of BAE cells on type I collagen in response to B16F10 CM. This assay represents a new model system to study tumour-induced angiogenesis *in vitro*.

**Keywords**: angiogenesis; chemotaxis chamber; bovine aortic endothelial cells; Matrigel; basic fibroblast growth factor

Angiogenesis is a primary requisite for progression of malignant solid tumours (Folkman, 1992). During tumour growth and metastasis, endothelial cells proliferate, degrade the surrounding basement membrane and migrate into the stroma. Finally, they differentiate, giving rise to new vessels (Blood and Zetter, 1990) which are crucial for nutrient delivery to tumours.

The interaction of endothelial cells with their microenvironment, especially with extracellular matrix components, plays an essential role during the angiogenic process (Ingber, 1992). It has been shown that basement membrane proteins, such as laminin (Grant *et al.*, 1989) and type IV collagen (Ingber and Folkman, 1989) among others, directly induce the formation of capillary-like structures (CLS) from endothelial cells. In several ways tumour cells themselves can also promote angiogenesis, e.g. by secretion of angiogenic factors, protease activation or macrophage stimulation (Folkman and Shing, 1992).

*In vivo* models, such as the chorioallantoic membrane assay or the rabbit cornea implant technique, are widely used for studying angiogenesis. These, however, do not permit evaluation of whether an angiogenic factor or an antiangiogenic molecule is acting directly on endothelial cells or indirectly via neighbouring cells, such as inflammatory cells and/or fibroblasts. Experimental approaches that reproduce the tumour-induced angiogenic process *in vitro* will help to study the molecular mechanisms involved in the interaction between tumour cells and endothelial cells during angiogenesis. Since the first description of maintenance of long-term cultures of capillary endothelial cells by addition of tumour cell conditioned medium (CM) (Folkman *et al.*, 1979), several *in vitro* angiogenesis assays have been described (reviewed in Auerbach *et al.*, 1991). These assays, such as the capillary formation within collagen or gelatin gels (Montesano and Orci, 1985; Pepper *et al.*, 1992), or the migration of endothelial cells in the silicon template compartmentalisation technique (Augustin-Voss and Pauli, 1992), permit the analysis of the effect of angiogenic factors or tumour cell CM on individual angiogenic steps.

We have developed a modified chemotaxis chamber assay that allows simultaneous study of the different steps of the overall angiogenic process: in this assay endothelial cells stimulated by tumour cell CM degrade basement membrane Matrigel, migrate towards the angiogenic stimulus and form CLS on type I collagen. This assay represents a useful tool for the study of factors involved in tumour-induced angiogenesis and for the characterisation of specific inhibitors of this process.

Correspondence: M Aracil
Received 3 August 1994; revised 14 November 1994; accepted 15 November 1994

### Materials and methods

### *Cell lines and culture conditions*

Bovine aortic endothelial (BAE) cells and the B16F10 murine melanoma cell line were obtained from Farmitalia Carlo Erba (Nerviano, Italy). B16F0 murine melanoma and MDA-MB-231 human breast carcinoma cell lines were obtained from the American Type Culture Collection (Rockville, MD, USA). Cell lines were cultured at 37°C, in a 5% carbon dioxide atmosphere, in Dulbecco's modified Eagle medium (DMEM) (Gibco, UK) (BAE cells, B16F0 and MDA-MB-231) or RPMI-1640 (Flow, UK) (B16F10), supplemented with 10% fetal calf serum (FCS) (Seralab, UK), 2 mM glutamine (Seralab, UK), 100 IU $ml^{-1}$ penicillin and 0.1 mg $ml^{-1}$ streptomycin. Cell lines were routinely checked by the Gen-Probe rapid detection system (Gen-Probe, San Diego, CA, USA) for mycoplasma contamination.

### *Reagents*

Basement membrane Matrigel and type I collagen were purchased from Collaborative Biomedical Products (Bedford, MA, USA). Human recombinant basic fibroblast growth factor (bFGF) was obtained from Farmitalia Carlo Erba. Suramin was purchased from Bayer (Leverkusen, Germany). Anti-mouse laminin and anti-human bFGF antibodies were obtained from Collaborative Biomedical Products.

### *Production of tumour cell CM*

Approximately $4 \times 10^6$ B16F0, B16F10 or MDA-MB-231 tumour cells were grown to subconfluency in culture medium. After washing three times with phosphate-buffered saline (PBS), serum-free medium was added. Three days later CM was harvested, centrifuged for 5 min at 500 *g*, passed through a 0.22 μm pore size filter and stored at −20°C until use. As the growth rate of cell lines was different, conditioned media were conveniently diluted to a similar number of cells per volume at the time of harvesting in order to reduce the variability in the concentration of putative factors secreted into the medium.

**Figure 1** (**a**) Scheme of a Transwell cell culture chamber used in the tumour-induced angiogenesis assay. Each well contains a Transwell insert (I) with a polycarbonate membrane (M); the insert delimits an upper chamber (U) and a lower chamber (L). (**b**) Magnification of the separation between both chambers: A, BAE cells in the upper chamber; B, Matrigel coating the upper side of the filter; C, 8 μm pore polycarbonate filter; D, type I collagen coating the bottom side of the filter (in some experiments type I collagen was replaced by Matrigel); E, tumour cell CM in the lower chamber (in some experiments CM was replaced by bFGF).

### In vitro *tumour-induced angiogenesis assay*

Polyvinylpyrrolidone-free polycarbonate filter Transwell inserts (6.5 mm diameter) with 8 μm pores (Costar, Cambridge, MA, USA) were used for the assay. Matrigel (50 μl from a 250 μg ml$^{-1}$ dilution in cold PBS) was applied to the upper surface of each filter and dried at room temperature under a hood. The underside of the filter was then coated with type I collagen (50 μl from a 250 μg ml$^{-1}$ dilution in 0.02 M acetic acid); in some experiments Matrigel (50 μl from a 750 μg ml$^{-1}$ dilution in cold PBS) was used instead of type I collagen. Tumour cell CM (600 μl) was added to the lower chamber. Approximately $10^5$ BAE cells (100 μl) were seeded in the upper chamber in DMEM supplemented with 0.2% bovine serum albumin. After incubation for 72 h at 37°C in a 5% carbon dioxide atmosphere, cells on the upper side of the filter were removed with cotton swabs. Filters were then stained with haematoxylin–eosin or with a Giemsa-modified staining method. The formation of CLS was assessed under the microscope and compared with controls having non-conditioned culture medium in the lower chamber.

### Results

### *Tumour cell-induced formation of CLS in BAE cells*

In order to study whether tumour cell CM could induce the formation of CLS in BAE cells, these were added to the upper compartment of Transwell cell culture chambers, and CM from either B16F0 or B16F10 cells, murine melanoma cells with different metastatic capacity (Fidler, 1973), was

**Figure 2** Formation of CLS by BAE cells in the tumour-induced angiogenesis assay. BAE cells were seeded in the upper chamber and after 3 days incubation the development of CLS on the bottom of the filter was evaluated. BAE cells formed CLS on type I collagen in response to B16 F10 CM (**a**), but not to B16F0 CM (**b**). When the type I collagen coating of the bottom side of the filter was replaced by Matrigel, BAE cells formed CLS also in the presence of B16F0 CM (**c**). BAE cells also formed CLS on type I collagen when tumour cell CM was replaced by 10 ng ml$^{-1}$ bFGF (**d**). Giemsa staining of the bottom side of the Transwell inserts is shown in all photomicrographs; filter pores appear as white circles. Original magnifications × 100; inserts in **a** and **b**, × 10.

added to the lower chamber; the filters of the Transwell inserts had been previously coated with Matrigel on the upper side and with type I collagen on the lower side (Figure 1). The amount of Matrigel (12.5 μg) was sufficient to create a basement membrane-like barrier but was unable to induce the formation of CLS by BAE cells (data not shown). When CM from the highly metastatic B16F10 cell line was present in the lower well, within 72 h BAE cells degraded Matrigel, migrated through the filter pores and differentiated into CLS on the type I collagen coating (Figure 2a). This CLS formation could also be observed using CM from the human metastatic breast adenocarcinoma cell line MDA-MB-231 (data not shown). In contrast, when CM of the less invasive B16F0 cell line was used, no CLS were formed on type I collagen (Figure 2b). However, when the lower coating of the filters contained Matrigel (37.5 μg) instead of type I collagen, no differences were observed between B16F10 (data not shown) and B16F0 CM (Figure 2c) and CLS were formed in both cases. Formation of CLS on type I collagen or Matrigel required in any case the presence of tumour cell CM, since few BAE cells migrated through the Matrigel layer and no differentiation was observed when non-conditioned culture medium was added to the lower compartment of the chamber (data not shown).

**Figure 3** Effect of an anti-bFGF antibody (**a**), an anti-laminin antibody (**b** and **c**) and suramin (**d**–**f**) on the formation of CLS on type I collagen by BAE cells in response to B16F10 CM. CLS formation was studied using the tumour-induced angiogenesis assay. Formation of CLS was abolished when an anti-bFGF antibody was added to the lower chamber (**a**). An anti-laminin antibody partially inhibited the formation of CLS when added to the upper chamber (**b**), but it had no effect when added to the lower chamber (**c**). When 10 μM (**d**) or 100 μM suramin (**e**) was added to the lower chamber a dose-dependent inhibition of CLS formation by BAE cells was observed; however, this inhibition was not observed when 100 μM suramin was added to the upper chamber (**f**). Giemsa staining of the bottom side of the Transwell inserts is shown in all photomicrographs. Original magnifications × 100.

**Table I** Formation of CLS in response to tumour cell CM. Summary of the results

| *Chemoattractant* | *Bottom side coating of the filter* | *Additions*[a] | *CLS formation*[b] | *Figure*[c] |
|---|---|---|---|---|
| B16F10 CM | Type I collagen | – | + | 2a |
| B16F10 CM | Matrigel | – | + | NS |
| MDA-MB-231 CM | Matrigel | – | + | NS |
| B16F0 CM | Type I collagen | – | – | 2b |
| B16F0 CM | Matrigel | – | + | 2c |
| None | Type I collagen | – | – | NS |
| bFGF (10 ng $ml^{-1}$) | Type I collagen | – | + | 2d |
| B16F10 CM | Type I collagen | Anti-bFGF (L) | – | 3a |
| B16F10 CM | Type I collagen | Anti-laminin (U) | ± | 3b |
| B16F10 CM | Type I collagen | Anti-laminin (L) | + | 3c |
| B16F10 CM | Type I collagen | 10 $\mu$M suramin (L) | ± | 3d |
| B16F10 CM | Type I collagen | 100 $\mu$M suramin (L) | – | 3e |
| B16F10 CM | Type I collagen | 10 $\mu$M suramin (U) | + | NS |
| B16F10 CM | Type I collagen | 100 $\mu$M suramin (U) | + | 3f |

[a]L, lower chamber; U, upper chamber. [b]±, partial inhibition. [c]NS, results not shown.

*bFGF-induced formation of CLS*

To evaluate whether bFGF, one of the most potent angiogenic factors known, is responsible for the angiogenic effect exerted by B16F10 CM, a neutralising anti-bFGF antibody (100 $\mu$g $ml^{-1}$) was added together with the tumour cell CM; in this case the formation of CLS was abolished (Figure 3a). Moreover, capillary formation was induced on type I collagen when B16F10 CM was replaced by 10 ng $ml^{-1}$ bFGF (Figure 2d).

*Laminin-induced formation of CLS*

Laminin is one of the major constituents of Matrigel. To evaluate the role of this basement membrane protein in our *in vitro* tumour-induced angiogenesis model, in a separate set of experiments an anti-laminin antibody (100 $\mu$g $ml^{-1}$) was added to the upper chamber, thus coming into contact with the Matrigel coating the filter. A reduction in the formation of CLS on type I collagen was observed (Figure 3b) when compared to conditions without antibody (Figure 2a). Moreover, addition of the anti-laminin antibody to the lower chamber, and thus in contact with the type I collagen coating the underside of the filter, had no effect on the formation of CLS by BAE cells (Figure 3c).

*Inhibition of the formation of CLS by suramin*

The effect of the anti-angiogenic and anti-tumorigenic agent suramin was evaluated in our model system. Addition of non-toxic concentrations of suramin (10 and 100 $\mu$M) to the lower chamber throughout the incubation period resulted in a marked dose-dependent effect, preventing endothelial cells from migrating and differentiating on type I collagen in response to B16F10 CM (Figure 3d and e). This inhibitory effect was not noticeable when suramin was added to the upper compartment of the Transwell cell culture chambers (Figure 3f).

A summary of the results is shown in Table I.

### Discussion

Long-term cultures of capillary endothelial cells were firstly obtained by co-culture with tumour cell CM, thereby suggesting that tumour cells produce growth factors necessary for endothelial cell proliferation (Folkman *et al.*, 1979). Later, taking advantage of this fact, *in vitro* assays specific for single steps of the angiogenic process (migration, invasion, differentiation, proliferation of endothelial cells) were developed and were applied to study the regulatory mechanisms of angiogenesis and to screen for inhibitors (Auerbach *et al.*, 1991). Differentiation of mouse lung endothelial cells cultured on plastic has been achieved in the presence of Lewis lung carcinoma CM (Li *et al.*, 1991). Changes in DNA and RNA content and synthesis were also observed when BAE cells were placed in contact with CM from human astrocytoma cells (Silbergeld *et al.*, 1992). Thompson *et al.* (1991) observed the induction of endothelial cell chemotaxis and invasion through type IV collagen and Matrigel-coated filters, respectively, by adding AIDS-related Kaposi's sarcoma cell CM to the lower well of a Boyden chamber. A similar assay for endothelial cell invasion has been described by Murata *et al.* (1991) based on the use of Transwell chambers; in their system invasion through Matrigel is induced by B16B26 murine melanoma CM as chemoattractant.

On the other hand, systems that use co-cultures instead of tumour cell CM have demonstrated that cell-to-cell contacts between tumour and endothelial cells can also contribute to the formation of new vessels. Co-cultures of astroglial cells with bovine retinal endothelial cells result in the formation of capillary structures (Laterra and Goldstein, 1991). A model system for tumour angiogenesis, based on the co-culture of tumour cell lines with endothelial cells in Transwell cell culture chambers, has recently been described. Using this co-culture system, tubular morphogenesis by human omentum microvascular endothelial cells in type I collagen gels was shown to be induced by transforming growth factor alpha (TGF-$\alpha$)-producing oesophageal tumour cells (Okamura *et al.*, 1992) or keratinocytes (Ono *et al.*, 1992). BAE cells also form capillary structures in type I collagen when co-cultured with human glioma cell lines (Abe *et al.*, 1993); in this case a direct correlation between tubulogenesis of BAE cells and bFGF mRNA levels in glioma cells was demonstrated. These findings support the hypothesis that tumour cells directly control not only the proliferation and invasion steps, but also the differentiation of endothelial cells during tumour-induced angiogenesis.

In order to develop a system able to reproduce simultaneously the overall angiogenic process, i.e. tumour-induced endothelial cell invasion and differentiation, we modified the chemotaxis chamber assay (Albini *et al.*, 1987) in such a way that tumour cell CM induces the degradation of a Matrigel barrier by endothelial cells, their migration through it and their subsequent differentiation into CLS on a type I collagen layer. Although our tumour-induced angiogenesis assay shows homology to the co-culture system described by Abe *et al.* (1993), there is a substantial difference between the models. In Abe's model, BAE cells seeded on a type I collagen gel migrate into it and differentiate into capillary structures; this tubulogenesis inside the gel requires a proteolytic degradation of type I collagen involving the activation of latent collagenase by tissue-type plasminogen activator (Sato *et al.*, 1993). In our model system, type I collagen degradation takes place at the lower site of the filter, but an additional invasion step needs to take place in the Matrigel coating the upper side of the Transwell inserts. This

Matrigel coating mimics more closely the *in vivo* situation in which endothelial cells have to degrade the basement membrane, whereas the type I collagen coating of the underside of the filter creates an environment similar to the interstitial matrix of the surrounding connective tissue, appropriate for the endothelial cells to organise into capillary structures. In summary, in our tumour-induced angiogenesis model, formation of CLS by BAE cells requires not only the activation of latent collagenases, but also the activation of type IV collagenases and other matrix metalloproteinases required for the degradation of Matrigel constituents.

In our model system BAE cells formed CLS on type I collagen in the presence of CM from the highly metastatic melanoma cell line B16F10 (Figure 2a), but CLS formation did not take place when CM from the less invasive B16F0 cell line was used (Figure 2b). It is possible that B16F0 cells do not produce sufficient amounts of angiogenic factors to induce the migration and differentiation of BAE cells, in contrast to B16F10 cells. Nevertheless, the possibility that B16F0 cells produce TGF-$\beta$ or other factors antagonising the effects of bFGF cannot be ruled out.

Formation of CLS could be blocked by anti-bFGF antibodies (Figure 3a), suggesting a role for this angiogenic factor in the tumour-dependent induction of angiogenesis shown in our assay, although other factors can be involved as well. The lack of signal sequences in the bFGF gene argues against its secretion via classical mechanisms; however, its externalisation and release into the culture medium by novel mechanisms has been proposed (Mignatti and Rifkin, 1991). The blocking of the effects of B16F10 CM by anti-bFGF antibody in our assay could therefore be explained if bFGF was released by such yet undefined mechanisms. A similar effect of anti-bFGF antibodies on the conditioned media of glioma cell lines has been described (Abe *et al*., 1993).

When Matrigel is used instead of type I collagen for coating the lower side of the filters, CLS formation is induced even in the presence of B16F0 CM (Figure 2c), suggesting that a Matrigel constituent (probably bFGF) is triggering the migration and differentiation of BAE cells; nevertheless, the presence of a chemoattractant factor (either bFGF or others) in the tumour cell CM in the lower chamber is an essential requirement for our assay since neither migration nor differentiation of BAE cells occurs when non-conditioned culture medium is used.

Laminin and other basement membrane components have been described as modulators of angiogenesis *in vitro* (Grant *et al*., 1989; Ingber and Folkman, 1989). In our tumour-induced angiogenesis model anti-laminin antibodies did not completely block the formation of CLS but reduced their number significantly (Figure 3b). This is probably due to an inhibition of the attachment of BAE cells to laminin and the subsequent laminin-induced secretion of matrix metalloproteases (Turpeenniemi-Hujanen *et al*., 1986), thereby blocking the migration and invasion of BAE cells through the Matrigel layer. The incomplete inhibition of CLS formation by anti-laminin antibodies in our assay could mean that other Matrigel components (probably type IV collagen, among others) are also involved in these events.

In order to validate this assay as a tool for studying tumour-induced angiogenesis, and especially for testing inhibitors, we evaluated the effect of suramin as a potential inhibitor. The mechanism of the anti-angiogenic activity of suramin is not completely understood, but the interaction of suramin with heparin-binding growth factors, such as bFGF, has been reported (Stein, 1989), as well as the *in vitro* inhibition of the binding of several growth factors (such as bFGF, EGF, insulin-like growth factor I and platelet-derived growth factor) to their cell-surface receptors on endothelial cells. Moreover, suramin has been recenlty reported to inhibit each of the key control points of angiogenesis: endothelial cell migration, proliferation and production of proteases (Takano *et al*., 1994). In our *in vitro* model, suramin, when added to the lower chamber, inhibited in a dose-dependent manner CLS formation by BAE cells on type I collagen (Figure 3d and e); this is probably due to binding to bFGF present in B16F10 CM, thereby neutralising its biological activity. This inhibition of tubular morphogenesis by suramin was much lower when the drug was added to the upper compartment (Figure 3f) than when it was added to the lower compartment of the Transwell cell culture chamber. This differential effect of suramin could be the result of its binding to heparan sulphate proteoglycans, one of the major components of Matrigel, thereby sequestering the drug and reducing its effective concentration; in this way, the ability of suramin to interact with growth factor receptors on endothelial cells and to diffuse through the filter, blocking bFGF in the lower chamber, would be greatly reduced.

In conclusion, in this *in vitro* model system we are able to study in combination the different steps that could be assayed separately with other previously described *in vitro* angiogenesis assays, i.e. basement membrane degradation (Murata *et al*., 1991), endothelial cell migration towards angiogenic factors (Augustin-Voss *et al*., 1992) or capillary formation within three-dimensional collagen gels (Pepper *et al*., 1992; Abe *et al*., 1993). This system reflects more closely the *in vivo* situation, and therefore we think that our tumour-induced angiogenesis model represents a valuable tool for the study of molecular interactions during the angiogenic process.

### References

ABE T, OKAMURA K, ONO M, KOHNO K, MORI T, HORI S AND KUWANO M. (1993). Induction of vascular endothelial tubular morphogenesis by human glioma cells. A model system for tumor angiogenesis. *J. Clin. Invest.*, **92**, 54–61.

ALBINI A, IWAMOTO Y, KLEINMAN HK, MARTIN GR, AARONSON SA, KOZLOWSKI JM AND MCEWAN RN. (1987). A rapid in vitro assay for quantitating the invasive potential of tumor cells. *Cancer Res*., **47**, 3239–3245.

AUERBACH R, AUERBACH W AND POLAKOWSKI I. (1991). Assays for angiogenesis: a review. *Pharmacol. Ther*., **51**, 1–11.

AUGUSTIN-VOSS HG AND PAULI BU. (1992). Quantitative analysis of autocrine-regulated, matrix-induced, and tumor cell-stimulated endothelial cell migration using a silicon template compartmentalization technique. *Exp. Cell Res*., **198**, 221–227.

BLOOD CH AND ZETTER BR. (1990). Tumor interactions with the vasculature: angiogenesis and tumor metastasis. *Biochim. Biophys. Acta*, **1032**, 89–118.

FIDLER IJ. (1973). Selection of successive tumour lines for metastasis. *Nature (New Biol.)*, **242**, 148–149.

FOLKMAN J. (1992). The role of angiogenesis in tumor growth. *Semin. Cancer Biol*., **3**, 65–71.

FOLKMAN J AND SHING Y. (1992). Angiogenesis. *J. Biol. Chem*., **267**, 10931–10934.

FOLKMAN J, HAUDENSCHILD CC AND ZETTER BR. (1979). Long-term culture of capillary endothelial cells. *Proc. Natl Acad. Sci. USA*, **76**, 5217–5221.

GRANT DS, TOSHIRO KI, SEGUÍ-REAL B, YAMADA Y, MARTIN GR AND KLEINMAN HK. (1989). Two different laminin domains mediate the differentiation of human endothelial cells to capillary-like structures in vitro. *Cell*, **58**, 933–943.

INGBER DE. (1992). Extracellular matrix as a solid-state regulator in angiogenesis: identification of new targets for anti-cancer therapy. *Semin. Cancer Biol*., **3**, 57–63.

INGBER DE AND FOLKMAN J. (1989). How does extracellular matrix control capillary morphogenesis? *Cell*, **58**, 803–805.

LATERRA J AND GOLDSTEIN GW. (1991). Astroglial-induced in vitro angiogenesis: requirements for RNA and protein synthesis. *J. Neurochem*., **57**, 1231–1239.

LI L, NICOLSON GL AND FIDLER IJ. (1991). Direct in vitro lysis of metastatic tumor cells by cytokine-activated murine vascular endothelial cells. *Cancer Res*., **51**, 245–251.

MIGNATTI P AND RIFKIN D. (1991). Release of basic fibroblast growth factor, an angiogenic factor devoid of secretory signal sequence: a trivial phenomenon or a novel secretion mechanism? *J. Cell. Biochem.*, **47**, 201–207.

MONTESANO R AND ORCI M. (1985). Tumor-promoting phorbol esters induce angiogenesis in vitro. *Cell*, **42**, 469–477.

MURATA J, SAIKI I, MAAKABE T, TSUTA Y, TOKURA S AND AZUMA I. (1991). Inhibition of tumor-induced angiogenesis by sulfated chitin derivatives. *Cancer Res.*, **51**, 22–26.

OKAMURA K, MORIMOTO A, HAMANAKA R, ONO M, KOHNO K, UCHIDA Y AND KUWANO M. (1992). A model system for tumor angiogenesis: involvement of transforming growth factor-α in tube formation of human microvascular endothelial cells induced by esophageal cancer cells. *Biochem. Biophys. Res. Commun.*, **186**, 1471–1479.

ONO M, OKAMURA K, NAKAYAMA Y, TOMITA T, SATO Y, KOMATSU Y AND KUWANO M. (1992). Induction of human microvascular endothelial tubular morphogenesis by human keratinocytes: involvement of transforming growth factor-α. *Biochem. Biophys. Res. Commun.*, **189**, 601–609.

PEPPER MS, FERRARA N, ORCI L AND MONTESANO R. (1992). Potent synergism between vascular endothelial growth factor and basic fibroblast growth factor in the induction of angiogenesis in vitro. *Biochem. Biophys. Res. Commun.*, **189**, 824–831.

SATO Y, OKAMURA K, MORIMOTO A, HAMANAKA R, HAMAGUCHI K, SHIMADA T, ONO M, KOHNO K, SAKATA T AND KIWANO M. (1993). Indispensable role of tissue-type plasminogen activator in growth factor-dependent tube formation of human microvascular endothelial cells *in vitro*. *Exp. Cell Res.*, **204**, 223–229.

SILBERGELD DL, ALI-OSMAN F AND WINN HR. (1992). Induction of transformational changes in normal endothelial cells by cultured human astrocytoma cells. *J. Neurosurg.*, **75**, 604–612.

STEIN CA. (1989). Suramin: an anticancer drug with a unique mechanism of action. *J. Clin. Oncol.*, **7**, 499–508.

TAKANO S, GATELY S, NEVILLE ME, HERBLIN WF, GROSS JL, ENGELHARD H, PERRICONE M, EIDSVOOG K AND BREM S. (1994). Suramin, an anticancer and antisuppressive agent, inhibits endothelial cell binding of basic fibroblast growth factor, migration, proliferation, and induction of urokinase-type plasminogen activator. *Cancer Res.*, **54**, 2654–2660.

THOMPSON EW, NAKAMURA S, SHIMA TB, MELCHIORI A, MARTIN GR, SALAHUDDIN SZ, GALLO RC AND ALBINI A. (1991). Supernatants of acquired immunodeficiency syndrome-related Kaposi's sarcoma cells induce endothelial cell chemotaxis and invasiveness. *Cancer Res.*, **51**, 2670–2676.

TURPEENNIEMI-HUJANEN T, THORGEIRSSON UP, RAO CN AND LIOTTA LA. (1986). Laminin increases the release of type IV collagenase from malignant cells. *J. Biol. Chem.*, **261**, 1883–1889.